# Structure and Optical Features of Micro/Nanosized Carbon Forms Prepared by Electrochemical Exfoliation

**DOI:** 10.1186/s11671-016-1770-5

**Published:** 2017-01-11

**Authors:** Sergii G. Nedilko, Sergiy Revo, Vitalii Chornii, Vasyl Scherbatskyi, Kateryna Ivanenko, Maksym Nedielko, Yurii Sementsov, Mykola Skoryk, Andrii Nikolenko, Victor Strelchuk

**Affiliations:** 1 Taras Shevchenko National University of Kyiv, Volodymyrska str. 64/13, 01601 Kyiv, Ukraine; 2 O. Paton Electric Welding Institute of NASU, Bozhenko str. 11, 03680 Kyiv, Ukraine; 3 Chuiko Institute of Surface Chemistry of NASU, General Naumov str. 17, 03164 Kyiv, Ukraine; 4 G.V. Kurdyumov Institute for Metal Physics of NASU, Acad. Vernadsky blv. 36, 03680 Kyiv, Ukraine; 5 NanoMedTech LLC, Antonovich str. 68, 03680 Kyiv, Ukraine; 6 Institute for semiconductor physics of NASU, 41, Nauky ave., 03028 Kyiv, Ukraine

**Keywords:** Carbon, Micro/nanosize, Morphology, Raman scattering, Luminescence

## Abstract

Micro/nanosized carbon materials were prepared by electrochemical exfoliation method in the forms of the colloids and thin films. Scanning electronic microscopy, optical and luminescent microscopy, and Raman scattering and luminescent spectroscopy were applied for characterization of materials. The wide photoluminescence band in the visible spectral region was observed for each of the samples. The shape of the photoluminescence band depends on excitation wavelength and on the size of the particles. At least two components with maxima at ~580 and ~710 nm can be distinguished in the photoluminescence spectra. The relations between the photoluminescence properties and morphology of the samples have been described and discussed.

## Background

Various types of carbon materials (carbon nanotubes and quantum dots, graphene, graphene oxide, etc.) are intensively studied since they are interesting from a viewpoint of both application and science. A science and technology roadmap that was proposed recently by Ferrari and co-workers [1] outlines the main perspectives of the study and application of graphene and its derivatives. The mentioned roadmap can be applied to the study of most micro/nanosized carbon materials (MNCM) as well. In the mentioned work, authors supposed that high-brightness luminescent elements can be elaborated on the base of graphene and graphene oxide. Such elaboration of carbon-based luminescent devices is of high importance because they can overcome known disadvantages of currently used lighting devices (high toxicity of the components, high price of rare-earth elements, low energy efficiency, etc.).

There are two important problems that must be solved at the current stage of carbon materials luminescence study: (1) relatively high price of the large quantity and high-quality MNCM production and (2) low intensity of the MNCM luminescence (increasing the luminescence intensity of MNCM is highly desirable). The first problem attracted great attention, and many methods of carbon nanostructure production were proposed (mechanical and chemical exfoliation, chemical vapor deposition, arc discharge, etc.) [2–9]. We used here electrochemical exfoliation method since it is cost-effective and it allows production of MNCM in large quantity [8].

Carbon nanostructures can reveal intensive luminescence. Thus, photoluminescence (PL) have been found in carbon nanotubes and carbon nanoparticles, dubbed carbon quantum dots and carbon dots [10–12]. Mono- and multilayer graphene due to the absence of band gap show only hot-carrier luminescence [13–15], but graphene oxides reveal intensive luminescence and its characteristics depend on preparation and treatment procedures [16–19]. At the same time, lack of data about photoluminescence properties of various MNCM is evident and determination of the MNCM luminescence origin requires further studies.

In this work, optical, luminescent, and scanning electron microscopy, Raman scattering spectroscopy, chemical element analysis, and luminescent spectroscopy were applied in order to clarify the origin of micro/nanosized carbon materials luminescence.

## Methods

### The Samples

Two sets of samples were obtained for the study—colloid carbon materials and carbon films on silica substrates. Colloids were prepared by electrochemical exfoliation method. The thermally expanded graphite was used as carbon source, and liquid KOH solution was used as electrolyte. In the result, homogeneous colloids consisting of MNCM particles (flakes) in alkali solution were obtained. Any precipitates of dispersed particles on the bottom of the flasks were not observed for the colloids after 6 months. Starting colloid was taken for study as sample #1 (hereafter C1). Two other samples of the set were obtained by filtering of starting solution through ceramic filters with pore sizes of 100 and 1 μ (denoted as C2 and C3 samples, respectively). Other sets of the samples consisting of the MNCM solid films were deposited on silica substrate by means of the small amounts of the mentioned C1, C2, and C3 solution evaporation. Those samples are denoted hereafter as P1, P2, and P3. Evaporation took place in ambient air conditions at temperature 60 °C during 16 h.

### Equipment

Optical microscope OLYMPUS GX51 and luminescent microscope were used for the sample topology study. Profound characterization was performed by scanning electron microscope (SEM) Tescan Mira 3 LMU with a 20-nm electronic beam diameter during the measurements. Detector of the secondary electrons (InBeam) enhances spatial resolution up to 1 nm. Besides the SEM imaging, microelement analysis of various areas of the samples was also performed using the same SEM.

A Triple T64000 Horiba Jobin-Yvon spectrometer equipped with a quasi-confocal scanning microscope was used for the Raman scattering spectra measurements. The scanning and optical systems allowed the movement of the object at XYZ coordinates with 100 nm accuracy and collection information about light scattering with submicron spatial resolution. The Ar-Kr Spectra Physics 2018 laser with wavelength of incident light, *λ*_inc_ = 488 nm, was used for the measurements.

The PL emission and PL excitation spectra were measured using single-grating (1200 grooves/mm) registration monochromators MDR-23 (linear dispersion 0.5 mm/nm) and DFS-12 (linear dispersion 1 mm/nm) equipped with FEU-100 and FEU-79 photomultipliers, respectively. The MDR-2 single-grating (1200 grooves/mm, linear dispersion 0.25 mm/nm) and double-prism DMR-4 (dispersion is from 0.5 to 0.05 mm/nm) monochromators were used as exciting ones. The N_2_ laser (*λ*_ex_ = 337.1 nm), two diode-pumped lasers (*λ*_ex_ = 473 and 532 nm), and arc Xenon lamp were used as sources of PL excitation. The PL spectra were studied as a function of the exciting radiation wavelength and were carried on in the wide region of excitation and emission wavelengths (200–800 nm). All of the PL and the PL excitation spectra were corrected on system response. The sample temperature was near 300 K (RT). A portable microscope allowed us to collect luminescence light with spatial resolution of the solid sample surface. The sizes of selected areas were in the range ~20–40 μ.

## Results and Discussion

### Microscopy

**Fig. 1:**
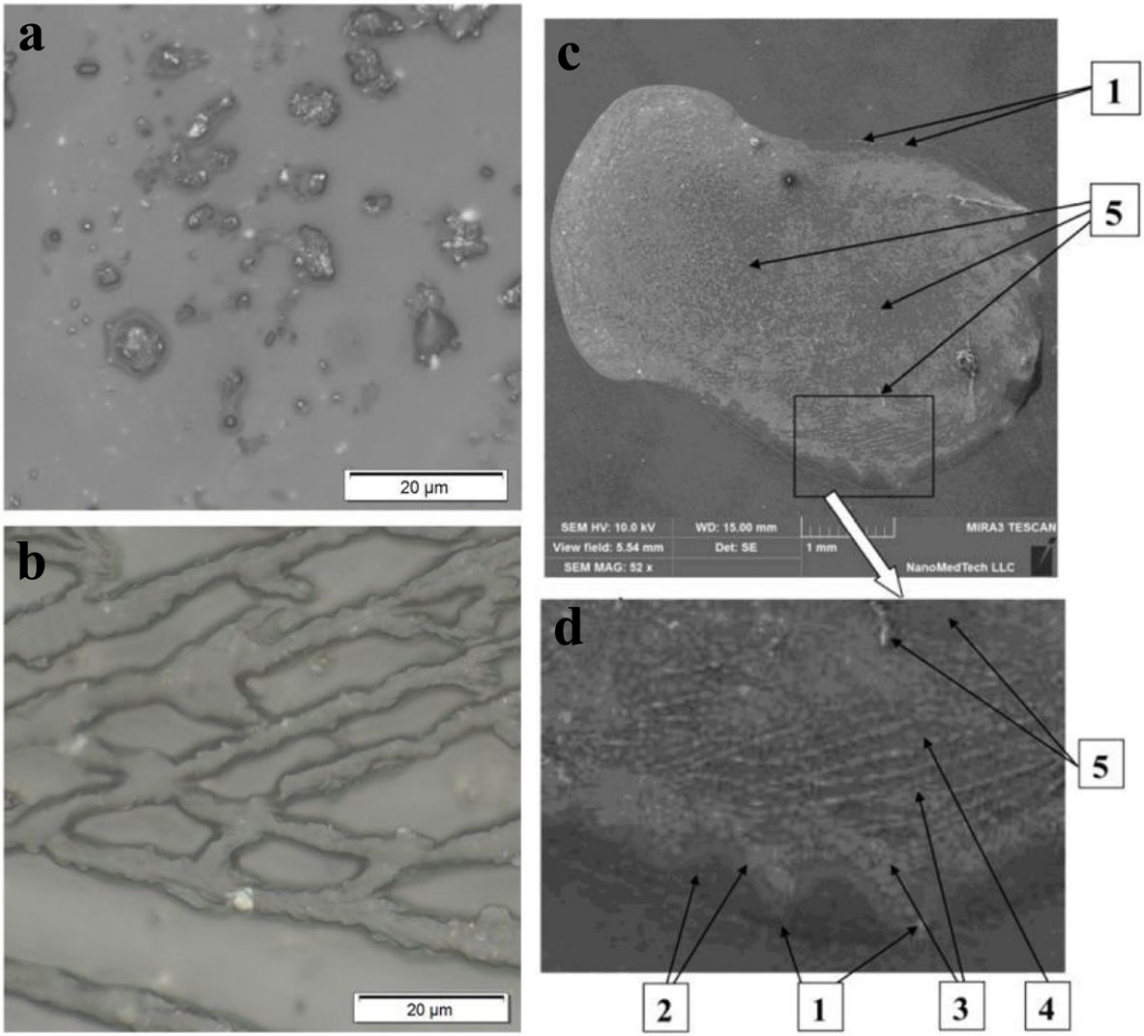
Optical microscopy (**a**, **b**) and SEM images (**c**, **d**) of the P1 sample. The various types of zones (1–5) of the sample are indicated by *arrows*. Some inclusions are shown also. *1* border line zone and inclusions, *2* tide zone and subzones, *3* dense net zone, *4* non compact net zone, *5* interior zone and inclusions

In the first stage of the solid sample study, they were characterized by optical microscopy. It was found that each of P1, P2, and P3 films is very inhomogeneous—separated particles are observed in the middle part of film and self-organized agglomerates of particles are present at the border area of the samples (corresponding images for P1 sample are shown in Fig. 1a, b). Mentioned inhomogeneity is an opportunity to conditionally select several regions on the film surface, which differ by the size and morphology of the carbon particle conformation.

More detailed study of the sample surface was performed by scanning electron microscopy. The SEM image of the whole P1 sample is presented in Fig. 1c, and its enlarged border part is shown in Fig. 1d. Five characteristic regions of different distribution of the MNCM particles were selected for the further study (see Fig. 1c, d).

**Fig. 2:**
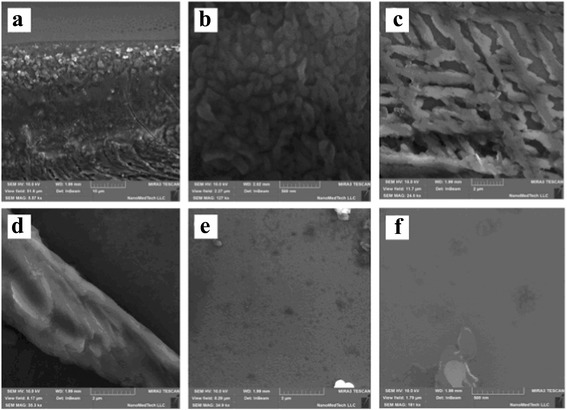
The detailed SEM images of solid sample P1 marked in the Fig. 1 zones: 1 (**a**), 2 (**b**), 3 (**c**), 4 (**d**), and 5 (**e**, **f**). Scale divisions are 10 μ (**a**), 2 μ (**c**–**e**), and 500 nm (**b**, **f**)

The SEM images of each selected region are shown in Fig. 2. The sizes of separated particles and their agglomerates in different parts of the sample are in the range from tens of nanometers up to tens of microns. The border part of the solid samples consists of two slightly different regions (hereafter zones 1 and 2). There are particles (the sizes are ≈1–2 μ) that cling close to each other with some inclusions of larger particles and wires in zone 1 (Fig. 2a). The next zone (zone 2) is located somewhat closer to the middle of the sample and contains submicron (100–400 nm) particles. The dense net of branch-like particles (width ≈0.5 μ, length >3 μ) is characteristic for zone 3. This net is located on the background of carbon layers formed by thin plates. Relatively long (width ≈3 μ, length >10 μ) isolated wires are observed at zone 4. Ensembles of different size (50–200 nm) separated particles are characteristic for zone 5 lying in the interior part of the films. Inclusions of the larger agglomerates are present somewhere within this zone too. According to the results of optical and scanning electron microscopy mentioned, selected regions of the P1 film differ by density, shapes, and sizes of the MNCM particles.

**Fig. 3:**
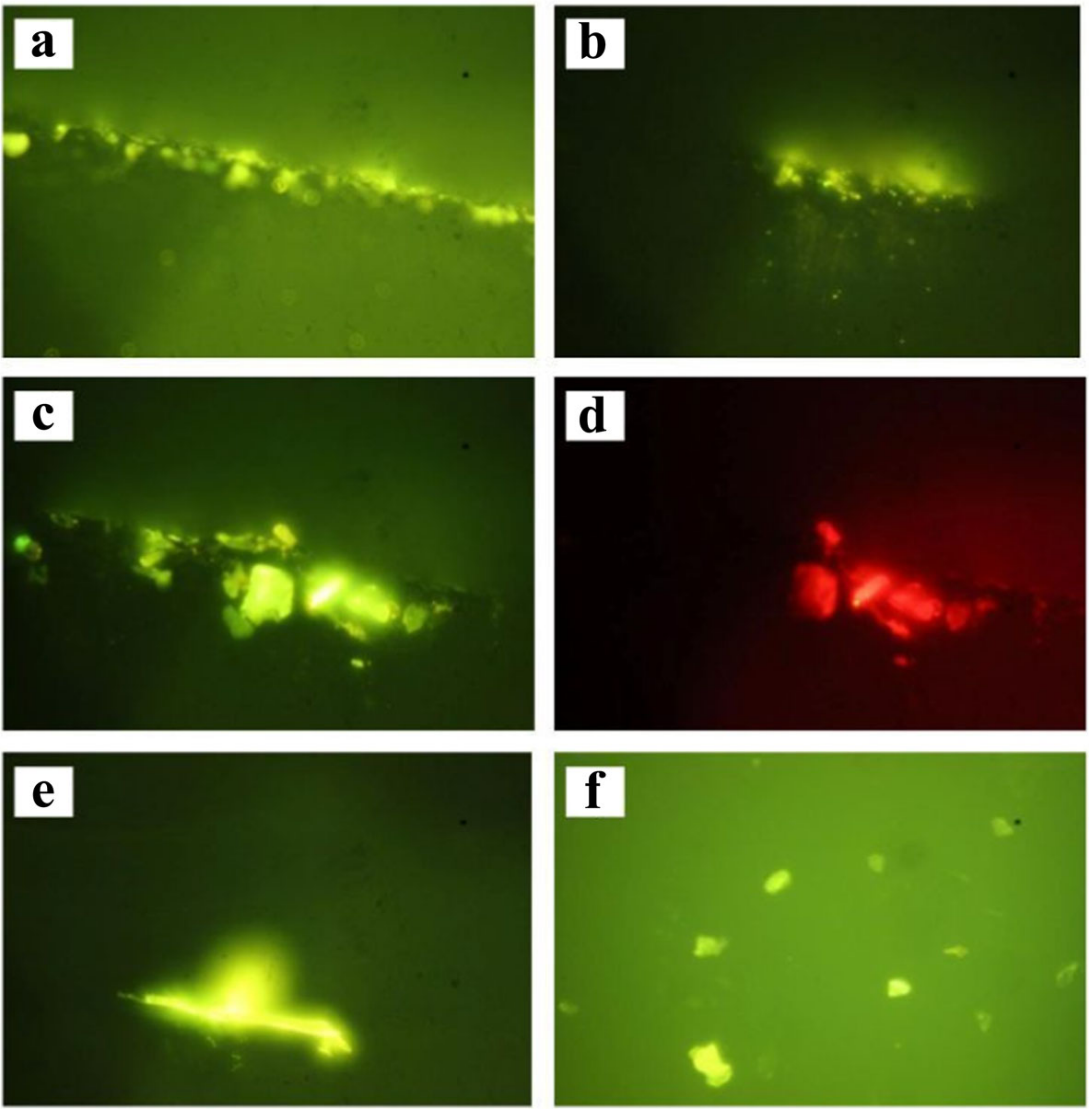
The luminescent images of the P1–P3 types of the samples. The horizontal size of the sample areas is 250 (**a**, **b**) and 25 μ (**c**–**e**). **a**–**d** The images demonstrate edge areas, while the **e**, **f** other images demonstrate interior of the samples. The *green* (**a**–**c**, **e**, **f**) and *red* (**d**) glass filters were applied under microscopic study

Some of luminescent microscopy images of the solid samples are presented in Fig. 3. Emission of the separated particles can be observed for all showed zones of the sample (Fig. 3a, b). At the same time, it is clearly seen that intensive emission is a property of some relatively large particles (Fig. 3c–f). Some of these objects reveal very intensive photoluminescence (Fig. 3c, e). The PL spectra are complex and at least consist of two (green and red) components.

So, photoluminescence is a property of separated particles and their agglomerates. However, the PL is weak in the regions where small and thin MNCM pieces are located, and it is much intensive for large particles and wires.

### Micro-Raman Spectroscopy

**Fig. 4:**
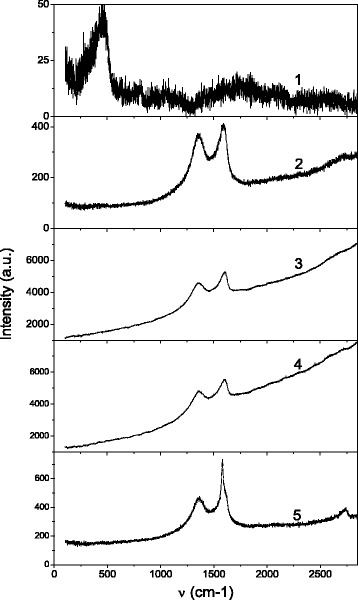
Raman scattering spectra taken at various areas of the P1 sample: *1* out of the sample; *2* zone 2; *3* zone 3; *4* zone 4: thick particle areas; *5* zone 5: thin particle areas. The sizes of monitoring areas are near 10 μ. *λ*_inc_ = 488 nm (2.54 eV); Pinc = 100 mW; *T* = 300 K

The Raman scattering spectra of the solid samples are shown in Fig. 4. The various regions of the sample surfaces were selected for Raman monitoring of the carbon materials. The region outside of carbon film (zone *out*) was also studied in order to consider possible influence of silica substrate on properties of the solid samples (Fig. 4, spectrum 1). Spectra 2–4 in Fig. 4 correspond to the regions where carbon layers have significant thickness (correspond to zones 2–4). Selected regions correspond to various types of the MNCM particle conformation and thickness which had been revealed previously by microscopy studies.

**Fig. 5:**
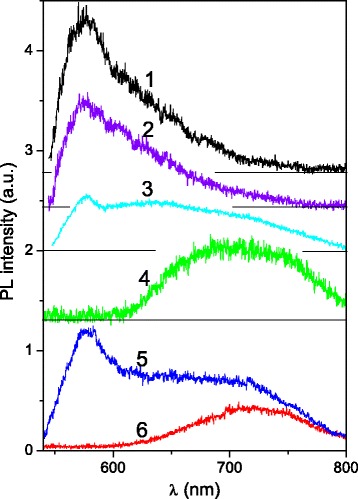
The PL spectra of the various samples (##1–4) of the P1 type: #1P1 (*1*), #2P1 (*2*), #3P1 (*3*), #4P1 (*4*) and the spectra taken by means of spatial selection of the emission from the surface of the P3 type sample (*5*, *6*): emission spectra for the areas that contain (*5*) and without bright fragments (*6*). *λ*_ex_ = 473 nm; *T* = 300 K

The Raman scattering lines of low intensity are observed at the low frequency range (<500 cm^−1^) for the zone out case. These lines correspond to the silicon oxide vibrations. Spectra 2, 3, and 4 are very similar to each other, despite their correspondence to different zones of the sample (zones 2, 3, and 4). The similarity of spectra indicates that particles of similar nature are present at the mentioned zones. The wide background on which the Raman lines are placed can be caused by the photoluminescence excited by incident light, *λ*_inc_ = 488 nm, (see Fig. 5 for comparison). Two intensive lines at 1357 and 1591 cm^−1^ (their full width at half maximum, FWHM, can be evaluated as 80–180 cm^−1^) are so-called the disorder-induced D band and Raman-allowed first-order G band, respectively [20–22]. Complex of low intensity band near ~2700–2730 cm^−1^ is the second-order (2D or G’) band of the zone-boundary phonons of carbon micro/nanostructures [22].

Spectrum 5 in Fig. 4 somewhat differs from spectra 2–4, but at the same time it contains the most of peculiarities inherent to Raman spectra of all studied zones of MNCM films. In particular, the D band at spectrum 5 has the shape, position, and FWHM similar to those on spectra 2–4. The G’ band has somewhat higher intensity than for zones 2–4 and up to four components of G’ band can de distinguished there. The most significant difference between spectra 2–4 and spectrum 5 is related with the G band region. The structureless wide G band is present in spectra 2–4 also, but additive intensive narrow line (FWHM ~15 cm^−1^) with peak position near 1580 cm^−1^ is observed there. The FWHM and position of the narrow line are typical for G band of graphene deposited on SiO_2_/Si [23]. This narrow line at 1580 cm^−1^ indicates that some amounts of graphene exist in zone 5 of MNCM solid films. Possibility to distinguish four components and shape of the G’ band allows us to assume a presence of multilayer graphene with four and or more layers [24, 25]. At the same time, similarity of spectrum 5 and spectra 2–4 indicates that some types of MNCM particles are inherent to all of studied zones in the solid samples.

A more detailed analysis of carbon nanostructures usually is performed by calculation of *I*_D_/*I*_G_ ratio (here, *I*_D_ and *I*_G_ are intensities of D and G bands, respectively). On the first stage of analysis, background (luminescent signal) of each Raman spectrum was subtracted. In addition, narrow band (approximated by Gauss curve with FWHM = 15 cm^−1^ and peak position at 1579.4 cm^−1^) was subtracted from spectrum 5. Calculated *I*_D_/*I*_G_ ratios are 0.91, 0.84, 0.94, and 0.84 for spectra 2, 3, 4, and 5, respectively. The well-known Tuinstra-Koenig relation, *I*_D_/*I*_G_ = C(*λ*)/*L*_a_ (where C(*λ*) is incident wavelength-dependent coefficient equal to 4.4 nm for *λ*_inc_ = 488 nm), allows us to estimate carbon particles sizes (*L*_a_) [26]. So, we found that carbon particles with sizes ~5 nm are at the studied zones 2–5 of the P1 sample. At the same time, we saw above that most of the particles at SEM images are of much larger sizes. Thus, we can assume that the large particles are really some agglomerates of MNCM particles of ~5-nm size. It is worth noting that similar Raman spectra were observed just for graphene oxide and graphite oxide nanoparticles [27, 28]. We suppose that agglomerates of graphene oxide and graphite oxide nanoparticles are inherent to all zones of the studied solid films, while multilayer graphene is present mainly at zone 5 of the films.

### Chemical Elements Analysis

Chemical elements analysis was performed for many specific points of the films using SEM microscope tools. It was found that C, Si, O, and K are the main components of the samples. Below we have discussed only distribution of the chemical elements on the MNCM particles at the selected earlier zones of solid samples. Corresponding results for the various points of the P1–P3 samples, where large and simultaneously thick particles and their agglomerates were situated, are noted in the Table 1. Obviously, the higher Si content indicates that particles have lower thickness and vice versa. Presented at the Table 1, data confirm our assumption about the existence of graphite oxide particles at zones 2 and 4. At the same time, there is low content of oxygen at zone 3 and consequently no high graphite oxide concentration can be expected. As for zone 5, chemical element analysis indicates a presence of mainly carbon materials, in particular multilayer graphene and graphite flakes.

**Table 1:** The content of some elements (mas. %) at the different zones of solid samples and accompanied luminescence intensity

		Si	C	O	K	Luminescence intensity
1	Zone 1, thick particles	1.27	31.50	36.89	29.75	Noticeable luminescence
2	Zone 2, small particles	7.11	30.41	31.18	30.25	No luminescence
3	Zone 3, dense “branches”	1.07	49.85	8.68	38.27	No luminescence
4	Zone 4, large particles	0.68	65.00	30.00	4.32	Noticeable luminescence
5	Zone 5, separated particles	0.96	84.76	7.41	6.86	Background luminescence
6	Zone 5, agglomerates (~5 μ)	0.65	64.00	12.00	21.60	Background luminescence
7	Zone 5, large particles	0.26	96.20	2.32	1.06	No luminescence

The luminescent microscopy and chemical element analysis allowed us to make some conclusions about the composition of luminescent MNCM particles (see Table 1). It looks like potassium has no influence on luminescence of studied zones of solid samples. Really, zone 1 is characterized by noticeable luminescence while zone 2 with almost the same potassium content reveals no luminescence. The regions with high carbon content and low oxygen content reveal only background luminescence. At the same time, region of thick particles with similar high content of C and O are characterized by intensive luminescence. An absence of luminescence for zone 2 (with highest Si content ≈7 mas. %) can be explained by the assumption that most of the oxygen are related to silicon oxides of substrate surface. Thus, intensive luminescence is characteristic of only those MNCM particles that are relatively large and contain both carbon and oxygen atoms.

### Luminescence Spectroscopy

Photoluminescence spectroscopy was applied to all the studied solid samples and starting colloid materials as well. The PL spectra of MNCM films P1 and P3 are shown in Fig. 5. Four different regions of sample P1, which contains carbon particles of various sizes, were chosen for luminescence monitoring. As Fig. 5 shows, the PL properties of solid sample P1 significantly depend on the region where luminescence spectrum was registered. There are at least two overlapping PL bands with maxima at *λ*_max_ ≈ 580 and 710 nm. This observation coincides with the results of luminescent microscopy where green and red luminescence were found for studied MNCM films. Both green and red luminescence were inherent to sample P3 (Fig. 5 curves 5 and 6) where sizes of particles do not exceed 1 μ. In general, the PL spectrum of the P3 sample (Fig. 5 curve 5) is similar to that of the P1 film. The regions of high brightness on the surface of the sample can be distinguished. The PL from these regions was measured using a microscopic portable device, and it is found that PL spectra consist of only one emission band with maxima at ~720 nm (or 1.72 eV). Interestingly, this value is very close to the band gap of graphene oxide cluster (1.7 eV) reported in [29] for saturated (meant high oxygen content) graphene oxide structure with chemical composition close to C_8_O_2_(OH)_2_. The same ratio of C to O content, 2:1, was obtained from chemical element analysis for zone 4, where noticeable luminescence was observed. That means that just a presence of the oxygen and/or hydroxyl groups determines luminescence of the large and thick MNCM particles. The luminescence mechanism in this case is related to radiation transition between the conduction and the valence bands of saturated graphite oxide structures. The bright luminescence of zone 1 where C:O ratio is 1:1 can be explained by the assumption that substantial part of oxygen is related to KOH remains.

On the other hand, the band gap of graphite oxides varies from zero up to several electronvolts depending on oxidation/reduction level [30, 31], and green luminescence of studied samples can be related to *unsaturated* graphite oxides. These results are in consistent with our data on chemical elements content at zone 5 where mostly background luminescence is observed. In fact, the C:O ratios are 6:1 and 12:1 for the regions of zone 5 where only background PL was found (see Table 1). The conclusion made above is in accordance with data of paper [17] where it was shown that the reduction of graphite oxide leads to blue-shift of luminescence spectra.

**Fig. 6:**
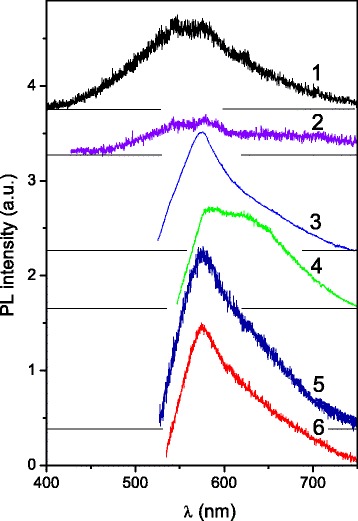
The PL spectra of the C1 (*1*–*4*), C2 (*5*), and C3 samples (*6*); *λ*_ex_ = 337.1 (*1*), 405 (*2*), 473 (*3*, *5*, *6*), and 532 nm (*4*); *T* = 300 K

In order to obtain the PL excitation spectra of MNCM samples, starting colloids C1, C2, and C3 were studied. Obviously, due to a higher particle concentration, the luminescence from colloids is much stronger than that from the solid films. The PL spectra of colloids were studied using laser excitations for comparison with the case of solid samples (Fig. 6). It is clearly seen from Figs. 5 and 6 that spectra of both colloid and films are similar for *λ*_ex_ = 473 nm. The luminescence of low intensity is also observed in *blue-green* spectral region (420–500 nm) under short wavelength excitation (337.1 and 405 nm). The shape and peak position, *λ*_max_, of the PL bands depend on the *λ*_ex_ as it was reported for various carbon colloids [10, 32–35].

**Fig. 7:**
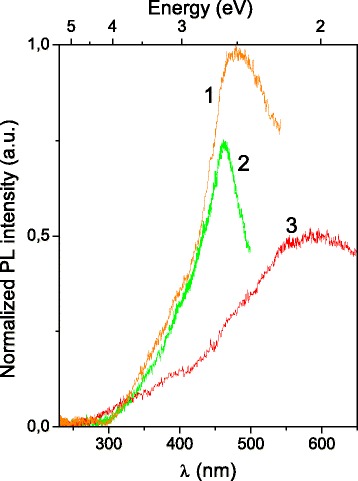
The PL excitation spectra for the C1 sample. *λ*_reg_ = 580 (*1*), 530 (*2*), and 700 nm (*3*). *T* = 300 K

The PL excitation spectra are shown in Fig. 7. Observation of a red-shift of excitation band maxima with increasing monitoring wavelengths is typical for carbon materials (see, e.g., [19]). The difference in band shape indicates that there are continuous (from a viewpoint of electronic band structure) sets of luminescent carbon structures, namely, graphite oxide particles with various reduction levels. The more detailed information about excitation mechanism in studied structures requires submicron separation of the particles we suppose.

## Conclusions

The colloid carbon systems obtained via electrochemical exfoliation from thermally expanded graphite possess luminescence properties. Their PL spectra are complex, where at least two components, green-yellow and red, can be selected. Importantly, the method applied allows a producing of the luminescent carbon micro/nano materials in large quantities without any additional treatment.

Studied micro/nano carbon materials consist of different size particles. The larger particles reveal characteristics of graphite oxides, and the smallest ones possess some characteristics of multilayer graphene. The small particles show a background luminescence, while the larger particles are able to reveal intensive photoluminescence. The luminescence effectiveness depends on oxygen group content. The most intensive photoluminescence was observed from particles where C to O ratio is equal to 2:1.

## Abbreviations

FWHM: Full width at half maximum
MNCM: Micro/nanosized carbon materials
PL: Photoluminescence
SEM: Scanning electron microscopy
